# Genome Sequence of *Serratia plymuthica* V4

**DOI:** 10.1128/genomeA.00340-14

**Published:** 2014-05-15

**Authors:** S. Cleto, G. Van der Auwera, C. Almeida, M. J. Vieira, H. Vlamakis, R. Kolter

**Affiliations:** a Department of Microbiology and Immunobiology, Harvard Medical School, Boston, Massachusetts, USA; b University of Minho, Department of Biological Engineering, Braga, Portugal; c Genome Sequencing and Analysis Group, Medical and Population Genetics Platform, Broad Institute, Cambridge, Massachusetts, USA

## Abstract

*Serratia* spp. are gammaproteobacteria and members of the family *Enterobacteriaceae*. Here, we announce the genome sequence of *Serratia plymuthica* strain V4, which produces the siderophore serratiochelin and antimicrobial compounds.

## GENOME ANNOUNCEMENT

The most commonly studied member of the genus *Serratia* is the pathogenic *Serratia marcescens*, a producer of a red prodigiosin pigment (1). *Serratia liquefaciens* and *S. marcescens* are often associated with nosocomial infections that involve the colonization of medical devices (2, 3). Strains belonging to this genus are known to harbor diverse antibiotic resistance determinants (3–8).

Other species belonging to this genus have been described as being indirectly beneficial to humans because they produce highly potent antifungal and antimicrobial molecules. Such is the case of *Serratia plymuthica* strain HRO-C48, which was isolated from the rhizosphere of oilseed rape and produces the antifungal pyrrolnitrin (9, 10), and of *S. plymuthica* RVH1, which was isolated from a raw vegetable-processing line and produces the antimicrobial zeamine (11, 12).

*Serratia* sp. strain V4 (ZK4911) was isolated from a biofilm that formed on the pasteurizer plate in a sanitized milk-processing line (13). This strain was particularly interesting, as it was found to produce the siderophore serratiochelin (14) as well as a number of uncharacterized antimicrobial compounds and the recently characterized zeamine (11, 13).

Genomic DNA from *Serratia* sp. V4 was obtained from an overnight culture in LB medium (Merck) using the total DNA extraction DNeasy blood and tissue kit (Qiagen). The genome sequence was obtained by *de novo* sequencing using 454 technology (Genotech, South Korea), which resulted in 65 contigs with an average length of 85 kb (contig size range, 10 to 457 kb; *N*_50_, 220 kb). The relative order and orientation of the contigs were determined by comparison with whole-genome sequences available from other members of the genus, those of *Serratia* sp. strain AS12 (GenBank accession no. CP002774.1), *S. plymuthica* 4x13 (previously *Serratia odorifera* 4Rx13; GenBank accession no. CP006250), and *Serratia proteamaculans* 568 (GenBank accession no. CP000826.1), using the whole-genome alignment tool Mauve version 2.3.1 (15). Based on this scaffolding, we were able to close 52 gaps by PCR and primer walking, thus yielding 13 supercontigs.

The genome of *Serratia* sp. V4 is approximately 5.5 Mb long, with a G+C content of 56%, falling within the normal *Serratia* genus values (54 to 60%) (16). The genome of *Serratia* sp. strain V4 displays a high degree of similarity (98% nucleotide identity) with the genome of *S. plymuthica* 4Rx13, though *S. plymuthica* 4Rx13 lacks key gene clusters involved in the biosynthesis of nonribosomal peptides and polyketides. For example, it lacks genes involved in the synthesis of zeamine (17), as confirmed by BLAST analyses.

Digital DNA-DNA hybridization (genome-to-genome distance calculator; German Collection of Microorganisms and Cell Cultures [DSMZ]) confirmed that *S. plymuthica* 4Rx13 is indeed the closest relative of *Serratia* sp. V4 (18). Formula 2 (sum of all identities found in high-scoring segment pairs [HSPs] divided by the overall HSP length), which is preferred for incomplete genomes such as this one, gave a percentage of 96.41 for the likelihood of these being the same species. Thus, we are designating this strain *S. plymuthica* strain V4.

### Nucleotide sequence accession number.

The genomic sequence of *S. plymuthica* V4 has been deposited in GenBank under the accession no. CP007439.
